# Co-Design of a website for women with pelvic organ prolapse: A study protocol

**DOI:** 10.12688/hrbopenres.13742.3

**Published:** 2024-01-11

**Authors:** Maria-Louise Carroll, Catherine Doody, Cliona O' Sullivan, Carla Perrotta, Brona M Fullen

**Affiliations:** 1 University College Dublin, Dublin, Leinster, D04C7X2, Ireland; 2 UCD Centre for Translational Pain Research, Dublin, D04C7X2, Ireland; 3 Physiotherapy Department, Tipperary University Hospital, Clonmel, Co. Tipperary, E91VY40, Ireland

**Keywords:** pelvic organ prolapse, physiotherapy, co-design, health literacy, gynaecology

## Abstract

**Background:**

Despite high reported prevalence of pelvic organ prolapse (POP), women report difficulties accessing evidence-based and reliable information about the condition. Many rely on social media and other popular and highly visible internet platforms which have been found to contain poor quality information that is difficult for the average patient to understand. The aim of the study is to co-design an information website for premenopausal women with POP. The website design will be based on the Website Developmental Model for the Healthcare Consumer (WDMHC) framework.

**Methods:**

A four phase process will be utilised as per the WDMHC framework: 1) User, task and environmental analysis; 2) Functional and representational analysis; 3) Cognitive walkthrough, keystroke level model, heuristic testing; 4) Content based testing, expert testing and user-based testing.

Ethics approval has been obtained (LS-23-19-Carroll-Ful). Two groups of stakeholders will be recruited (i) patient group (ii) healthcare professional (HCP) group. Patient participants will be recruited from an online pelvic floor dysfunction (PFD) support group (n=950 members). A website designer and HCP stakeholders involved in the multidisciplinary team caring for women with POP will be invited to participate.

Both groups will participate in separate co-design online workshops. Focus group workshops will be video-recorded, transcribed and imported into NVivo. Themes and subthemes will be developed.

The website will be designed and disseminated to all participants for feedback. Cognitive walkthrough and heuristic testing will be undertaken. Following this, necessary modifications will be made to the website. Participants will then complete a modified System Usability Scale (SUS) and the eHealth Impact Questionnaire, while five HCPs will complete the DISCERN instrument.

**Conclusion:**

This study will inform the design and testing of an information website for women with POP. The website design and content will be informed by patient and HCP stakeholder voices and the health literacy literature.

## Background

Female pelvic organ prolapse (POP) is defined by the International Urogynecological Association (IUGA) and International Continence Society (ICS) as a departure from normal sensation, structure, or function, experienced by the woman in reference to the position of her pelvic organs ^1^.

More than 30 million women worldwide are affected by POP ^2^, with some epidemiological research suggesting a rapid increase in prevalence of the condition in the next 25-30 years, particularly in high income countries ^3^.

It is associated with a range of physical symptoms, including a feeling of vaginal bulge, dragging, pain and other bladder, bowel, and sexual issues ^3^. These symptoms result in significant psychological and social impact including fear, regret, sadness hypervigilance and fear avoidance of certain activities ^4– 6^. These impacts appear to be compounded for women by a lack of knowledge about POP and difficulty sourcing reliable and evidence-based information about the condition ^3, 4^.

The provision of adequate information and counselling for women with POP has been highlighted as important for women to facilitate increased understanding of the condition, to promote active self-management where possible and increase shared decision-making between patients and their healthcare professionals (HCPs) ^4, 7– 11^.

Despite this, women have reported difficulty accessing evidence based, trustworthy and accurate information about POP ^12^. Moreover, women report HCPs as often lacking the time or expertise to provide adequate and detailed answers to their questions, or to offer health education or counselling when they present with symptoms or are diagnosed with POP ^4, 8– 10, 13^. The lack of accessibility to best practice information leads women to access other popular and highly visible web resources such as Wikipedia, Google and YouTube. However these are often reported as being difficult to read, biased, omitting important information or including misleading or incorrect information about the condition ^13– 21^.

Also a consideration is that most information on PFD accessible on the internet is at a reading level that is too difficult to understand for the average person ^22^. Health literacy research has consistently found that health information on PFD and POP is well above the recommended reading level of grades 5–8 recommended by the US National Institute of Health (NIH) and Centre for Disease Control, the Joint Commission and the American Medical Association (AMA) ^23, 24^.

Expert-designed health information is often overly generic and not adequately aligned with the abilities, preferences and life situations of the audience. It is suggested that participatory design of health information may improve health literacy and health promotion interventions ^25^.

This study is informed by our previous work in this field; utilising a systematic review to investigate the biopsychosocial profile of women POP and a qualitative investigation of the lived experience of women with POP ^4^. Lack of information about POP (among both women and HCPs) and high levels of anxiety and fear was highlighted in this research as a major problem for women. They reported significant concerns regarding inadvertently worsening their condition by undertaking activities they perceived as risky. As a consequence, women described fear avoidance behaviours, particularly related to physical activity ^4^.

Therefore, we propose a participatory action research framework to inform the development of a national information website for women with POP and other stakeholders.

## Study aims

To develop and design a national evidence-based information website for women with POP using a participatory action research approach.

## Study design framework

The study design will be based on the applicable principles of the Website Developmental Model for the Healthcare Consumer (WDMHC) ^26^. This model is a comprehensive, user-centred framework for the design and development of healthcare websites and has been shown to result in the delivery of a product that is likely to meet the expectations of end users and have minimal usability issues during end user testing ^27, 28^.

**Figure 1.**
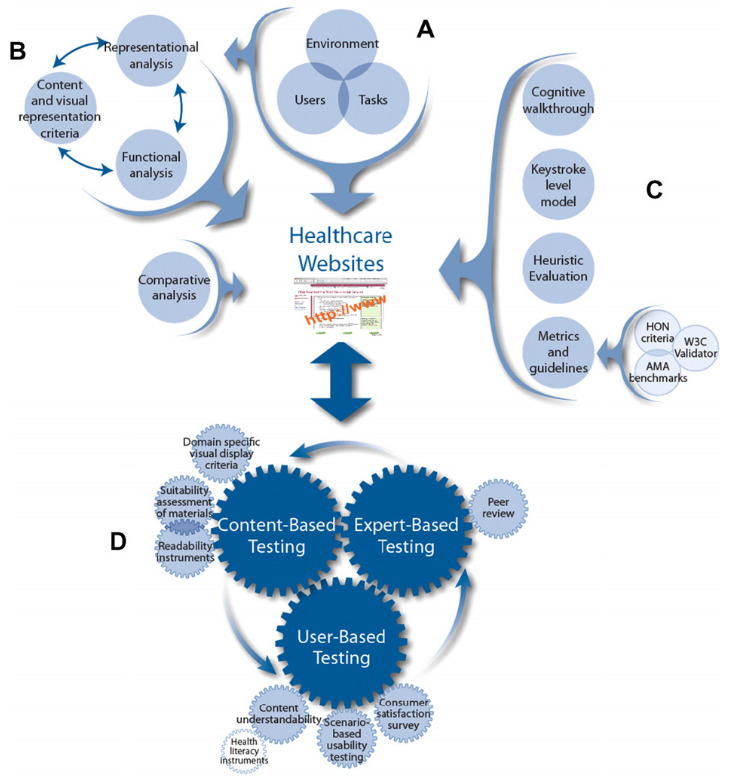
Website Development Model for the Healthcare Consumer.

The WDMHC model ( Figure 1) provides recommendations for website design in three main areas (i) user/environmental/task/functional analysis (part A), (ii) visual-graphic representation and (iii) comparative analysis (part B). Parts C and D provide recommendations for (i) usability, (ii) heuristic testing and content and (iii) expert and user based testing.

Incorporating the Noorbergen guidelines, which aim to address commonly encountered challenges in co-design during each step of the WDMHC will ensure a robust, standardised approach to the co-design process. They include: consideration of the circumstances of the health promotion context; consultation of behaviour change literature; engaging co-design facilitators with an understanding of the problem; immersion in the underlying health context to identify and understand stakeholders; identification of potential post-design advocates; ‘real world’ feasibility testing and analysis of intervention impact ^26, 29^. This is to ensure that the resulting intervention prioritizes and includes the perspectives of all major stakeholders, in particular the target end-user (women with POP), is evidence-based, pragmatic and applicable to multiple health service areas.

## Methods

### Step 1: User, Task & Environmental Analysis

***1.1 Contextual Enquiry***

The study will be informed by our earlier research exploring the biopsychosocial profile and lived experience of premenopausal Irish women with POP seeking healthcare ^4^. Additionally, end user characteristics such as age, race, ethnicity, computer skill level and information goals, as well as environmental analysis including physical, social and cultural context will be explored using focus group questions, the current literature and clinician experience. Task and functional analysis will be informed by the literature and patient and clinician experience with additional input from a website design expert.

***1.2 Forming a Planning/Development Team***

The development team will include four physiotherapists, including a specialist pelvic health physiotherapist with fifteen years of experience in the field, and three with backgrounds in qualitative research and health literacy, a GP with a background in research and women’s health and an obstetrics and gynaecology consultant. A website designer will also be included on the research team.

***1.3 Ethics approval***

Ethical approval has been granted by the University College Dublin Human Research Ethics Committee (HREC) (REF: LS-23-19-Carroll-Ful). The study will be performed in accordance with the Declaration of Helsinki.

***1.4 Participant Selection***

Two groups of stakeholders will be recruited (i) patient group (n=10 participants) (ii) HCP group (n=6 participants).

Patient inclusion criteria will be adult women (>18 years) with POP diagnosed and staged by a HCP, at least one year post-partum, premenopausal, not currently pregnant and able and willing to give informed consent. Sampling will be undertaken to ensure a range of POP stages (I-IV).

Relevant HCP stakeholders will include individuals from several involved disciplines including GPs, midwives, practice nurses/smear takers, gynaecologist/obstetricians and pelvic health physiotherapists.

**Recruitment**

Patient participants will be recruited from an online PFD support group (n=950 members). This group was set up by the principle investigator (MLC) on the social media platform Facebook in 2018 to provide a discussion forum and peer support for women with PFD.

Members will be invited to participate by an advertisement on the support group website outlining the study and patient inclusion criteria. Potential participants can contact the researcher (MLC) by phone or email. The participant information leaflet will be provided and after allowing a period of 1 week reflection the participant will be contacted by email by the researcher MLC to confirm their participation and provide the consent, demographic and eHealth Literacy questionnaires.

HCPs will be purposefully recruited by the development team including general practitioners (GPs), midwives, practice nurses/smear takers, gynaecologist/obstetricians and pelvic health physiotherapists. The development team will invite these HCPs to participate. HCPs will also be given 1 week to consider their participation and then contacted by the researcher (MLC) and if agreeing to participation, the HCP consent, demographic and eHealth Literacy Questionnaires will be provided.

***1.5 Development of Preparatory Materials***

Prior to the workshop patient participants will complete:

(i) a consent form
(ii) a patient demographic form
(iii) eHealth Literacy Questionnaire

HCPs will complete:

(i) a consent form
(ii) a HCP demographic form detailing their clinical experience and exposure to management of POP
(iii) eHealth Literacy Questionnaire.

Preparatory materials will be developed by the research team including:

(i) Participant instruction leaflets for using the Zoom & JamBoard platforms
(ii) Questions for workshop discussions
(iii) Patient vignettes to promote participant exploration of the ‘ideal’ scenario rather than a problem-solving approach
(iv) Details of other publicly available websites that will be discussed for comparative analysis to the proposed website

***1.6 Technology Tests***

The online focus groups will be held on Zoom and the JamBoard platform, and will be tested with participants prior to the workshops to ensure familiarity with both platforms.

### Step 2: Functional & Representational Analysis & Creation of the Prototype

***2.1 Framing the Issue***

This study will build on previous research by the authors exploring Irish women’s experiences of accessing care for POP and their recommendations for improvements. The development team have also conducted a systematic review exploring the biopsychosocial profile of Irish women with POP and qualitative research examining the lived experience of POP from a biopsychosocial perspective ^4^.

Access to information about their condition as an important facet of self –management and shared decision making was highlighted by the participants in this research. One researcher has extensive clinical experience working in the area of women’s pelvic health which will contribute to an understanding of the various stakeholder perspectives.

***2.2 Defining the goal of the intervention***

Patients and HCPs will be sent pre-workshop packs (see *Extended data*) for questions related to the workshop activities). These will include aims of the workshop and details of activities that they will participate in during the workshop. Content will include background information on POP and findings from qualitative research, details of similar publicly available information websites for comparative analysis, At the beginning of each workshop the aim of the intervention will again be explained and clarified where necessary.

***2.3 Generating ideas***

The co-design process will consist of separate patient and HCP workshops. This aims to eliminate any potential perceived power imbalance which may affect patient or HCP ability to share their views within their groups. Workshops will be carried out online, via Zoom. Zoom has been chosen as a platform with which many people are familiar and where interviews can be recorded and auto-transcribed.

Online workshops are favoured because of their accessibility, reducing the need for participants to travel, take time off work or source childcare to attend. This format will also allow the inclusion of a range of participants from across the country.

**Figure 2.**
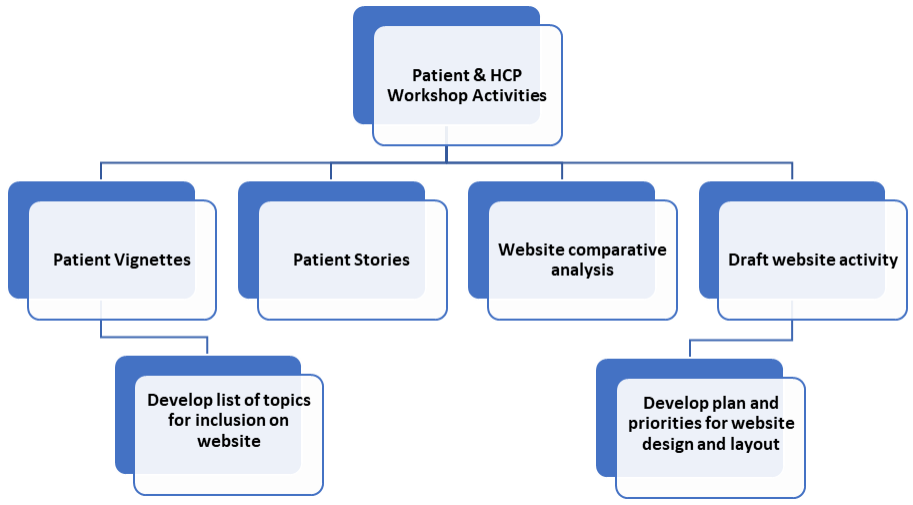
Planned activities and goals for patient and HCP workshops.

Workshop activities will aim to outline the ideal website content and design from both a patient and HCP perspective. Planned activities and goals for the workshops are shown in Figure 2 below.

**Patient Workshops**

Patient co-design workshops will be longer, reflecting the need to place patients at the centre of their care. They will run for approximately 2.5 hours and comprise of four activities; (i) semi-structured discussion in two smaller groups. During this discussion participants willing to share stories of their experiences and difficulties faced in the current state will be encouraged to do so; this will help the development team and participants in understanding what must change (see Appendix A at 10.6084/m9.figshare.22923290 for guiding questions). The development team will facilitate these conversations using questions designed to identify strengths, weaknesses and opportunities for improvement to the current state, leading to a clear overall concept for the website content, (ii) comparative analysis of similar websites (see Appendix C under *Extended data*), (iii) patient vignettes – this generative technique aims to imagine a future state where personas can receive the ideal resources and information. To distil details of this ideal future state, guiding questions will be used to direct group discussion (see Appendix B under *Extended data* ) (iv) draft layout design for website (see section 2.5 for questions)

**Healthcare Professional Workshops**

HCP workshops will run for a period of approximately one hour. The HCP session will consist of one group session and will use the same four activities as in the patient workshops. The same two patient vignettes will be used for patient journey mapping in order to observe differences in the HCP and patient perspective of the information needs of patients with POP.

After the workshops a list of topics for the website content will be compiled. The development team will consult the POP literature for evidence-based information under each topic heading. The development team will also use information from the comparative analysis and the draft layout workshop activities to inform the layout and design of the prototype website. The website developer will attend both the patient and HCP focus group workshops.

***2.4 Sharing ideas***

A sharing of ideas session bringing each group together to present the smaller group ideas will take place after the semi-structured discussion and patient vignettes activities. During this session the facilitator (a member of the development team) will also enquire about their thoughts on findings from qualitative research on women’s experiences of accessing care for POP and their recommendations for improvement. This information will be provided in the pre-workshop packs.

The sharing ideas session after the semi-structured discussion will further frame the issue for the development team and workshop participants, as well as highlighting challenges which may need to be overcome. The patient vignette is designed to encourage participant view of a future ‘ideal’ scenario rather than solutions to existing problems. The sharing of these vignettes from both a HCP and patient perspective may help to expose tacit knowledge or latent information needs that may be unconscious or difficult for participants to express in words ^27^.

The sessions will end with participants organising agreed topics into a draft layout design for the website.

***2.5 Comparative analysis to similar websites***

Comparative analysis will be carried out to similar websites during both the patient and HCP workshops. The details of these websites will be included in the pre-workshop packs and shared again during the workshops.

Participants will be asked several questions about the websites regarding the content: (i) what did you feel as regards the relevance of the content (ii) What did you think about the usefulness of the content? (iii) How reliable would you perceive the content of this website to be? (iv) Did the site contain all the information you were looking for? (v) Was there anything about the content of the website that you disliked? (vi) Is there any way you feel the content of the website could be improved upon?’ and design: (vii) ‘What are your thoughts on how the information on the website is organised? (viii) How easy or convenient did you find navigating the website? (ix) Where you satisfied with the website speed (how quickly it retrieved the information you were looking for)? (x) What is your opinion of the website’s layout? (xi) What did you think of the way information was presented (the format the information is given in, eg videos, multimedia)?

These questions are based on a hierarchical framework developed by Moustakis *et al*. which supports website quality assessment ^28^.

***2.6 Intervention Development***

**Requirements Translation/Focus Group Analysis**

The focus group workshops will be video-recorded, transcribed verbatim and imported into NVivo. The lead researcher (MLC) will complete a descriptive synthesis of the workshop activities and focus group transcripts. Developed themes and subthemes will be communicated to the focus group participants via email to ensure agreement with summary views.

Once themes and subthemes have been reviewed and approved by participants, the required elements of the website will be devised (content to be included, draft website layout, sorting the information into logical sections, the evidence-based POP literature all considered, using a health literate-sensitive approach). This work will inform the content and design for each section of the website by the development team including a website designer.

Priorities will be developed for the website design and action items and timelines assigned to each theme and subtheme. Tasks will then be assigned to the appropriate research team member. Focus group participants will be informed via email of the plans and timeline for the intervention design.

### Step 3: Cognitive Walkthrough & Heuristic Testing

Participants from the original focus group workshops will be invited to participate in testing of the website to ensure the usability, impact and quality of the end product aligns with the goals of the development team and study participants.

***3.1 Usability Testing & Cognitive Walkthrough***

Research has found that at least five participants are necessary in any usability testing cycle to detect up to 80% of issues ^30, 31^. Once the platform has been fully developed, two approaches will be used:

(i) Modified Usability Scale (SUS)
  Five participants from the patient focus group will be recruited to complete a modified SUS. This instrument has been validated as useful and robust and produces a single score, which can be compared against standardized acceptability scores ^32^.
(ii) Cognitive walkthrough testing
  This will be used to gather deeper insight into patient experience and usability of the intervention ^33, 34^. This will be done via Zoom with the tester sharing their screen through the Zoom platform while the researcher observes. These meetings are recorded. Tasks will be identified such as the seeking of health-related information or finding information about HCPs and steps for each task will be documented. Each task within the website will then be assessed using the standardized questions outlined by Taylor *et al.* ^35^: (i). What are the users’ goals?; (ii). Is the action obviously available?; (iii) Does the action or label match the goal?; (iv) Is there good feedback?

***3.2 Heuristic Evaluation***

Heuristic evaluation of the intervention on computer and mobile devices will be carried out in line with Nielsen’s ten usability heuristic principles by five development team members including a website designer ^36^. One patient and one HCP from the focus groups will also be asked to carry out heuristic testing. The areas of focus will include search, navigation, forms and data entry, information architecture, writing and content quality, trust and credibility, page layout and visual design.

The development team members will independently rate each website page using the following criteria: +1 (complies), -1 (does not comply), or 0 (partially complies). These reviewers will provide comments to justify their scoring. For items deemed not relevant, they will note ‘not applicable’. The development team will meet to discuss the ratings and reach an agreement regarding the items to be addressed before usability testing.

The results of the usability and heuristic testing will inform modifications to the website. Following these modifications content-based assessment, including expert-based and user-based testing will be carried out.

### Step 4: Content Based Testing

***4.1 Standards of Practice***

Standards of practice to ensure quality and credibility of internet health information have been established in many countries.

(i) The development of the study intervention will be informed by The Health on the Net Foundation Code of Conduct (HONcode) ^37^ While HONcode certification is no longer available, the HONcode principles which promote transparent and reliable health information online will be adhered to. For websites, it consists of eight principles, with detailed guidelines for each principle. It is used and approved by the Economic and Social Council and the WHO.

***4.2 Expert Based Testing***

(i) The DISCERN instrument ^38^ will be used by the HCPs involved in the HCP focus group. The 16 item instrument will peer-review the content of the website for quality, accuracy and reliability using the DISCERN instrument, a 16-item rating scale, to Once these steps are complete the prototype will be disseminated to the patient focus group participants who will be asked to complete a modified System Usability Scale (SUS) and the eHealth Impact Questionnaire. Following this, any necessary modifications will be made to the website.

***4.3 Readability***

It is recommended that patient educational information should not exceed the reading level of an 11 to 12-year old (or approximately 5th-8th grade level ^39– 42^. The website’s content will thus be assessed and adjusted as necessary to achieve an average Flesch-Kincaid grade level of between five and eight. The development team will also consult the National Adult Literacy Agency (NALA) Plain English Guidelines, as well as the Health Service Executive (HSE) Guidelines on Communicating Clearly Using Plain English With Our Patients and Service Users ^43, 44^.

***4.5 User Based Testing***

The same five patient participants recruited from the focus group will be asked to complete the eHealth Impact Questionnaire (e-HIQ) ^45^. This instrument assesses the impact of using websites containing health information. It consists of two distinct categories (eHIQ-Part 1 and eHIQ-Part 2). eHIQ-Part 1 is an eleven item scale assessing general attitude towards using the internet to access health information. eHIQ-Part 2 is a 26 item score assessing a participant’s experience of using a specific website.

Following content-based, including expert-based and user-based testing, modifications to the website will again be made as necessary.

## Study status

This study is currently at recruitment stage.

## Conclusions

Social media and the internet are ubiquitously used to search for and share medical information. According to the literature women are more likely to search online for diagnoses than men ^46^. Given the difficulties expressed by women with POP in obtaining reliable and complete information regarding their condition, this study aims to design and develop an evidence-based information website for this cohort. In the current project, there will be collaboration between patient users, health professionals, researchers and a website developer to develop a website based heavily on feedback from real patient users. The use of this participatory action research design aims to ensure a user-centred, reliable and evidence-based design.

## Data Availability

No data are associated with this article. Figshare: Appendices Co-design Protocol.docx. https://doi.org/10.6084/m9.figshare.22923290.v1 ^47^. Appendices Co-design Protocol.docx Data are available under the terms of the Creative Commons Attribution 4.0 International license (CC-BY 4.0).
