# Supplementary material for: Co-Design of a website for women with pelvic organ prolapse: A study protocol
Source: HRB Open Res. 2025 May 29;6:37. Originally published 2023 Jul 26. [Version 4] doi: 10.12688/hrbopenres.13742.4 (PMC10576183; doi:10.12688/hrbopenres.13742.4)
Supplement: Peer review history

# Reviewer report: Reviewer response for version 3

**DOI:** 10.21956/hrbopenres.15181.r37727

**Published:** 2024-01-27

**Authors:** Rohna Kearney, Rebecca Helliwell

**Affiliations:** 1 Manchester University NHS Foundation Trust, Manchester, UK; 2 Gynaecology, Manchester University NHS Trust, Manchester, Lancashire, UK

The authors have addressed the suggestions in our previous review. I have no further comments and wish them the best of luck in their study.

Is the study design appropriate for the research question?

Partly

Is the rationale for, and objectives of, the study clearly described?

Yes

Are sufficient details of the methods provided to allow replication by others?

Yes

Are the datasets clearly presented in a useable and accessible format?

Not applicable

Reviewer Expertise:

Urogynaecology, pelvic floor, self-management, pelvic floor imaging, obstetric childbirth injury

We confirm that we have read this submission and believe that we have an appropriate level of expertise to confirm that it is of an acceptable scientific standard.

# Reviewer report: Reviewer response for version 3

**DOI:** 10.21956/hrbopenres.15181.r37725

**Published:** 2024-01-27

**Authors:** Sara Cichowski

**Affiliations:** 1 Oregon Health & Sciences, Portland, Oregon, USA

I agree that the revisions address my concerns.

Is the study design appropriate for the research question?

Yes

Is the rationale for, and objectives of, the study clearly described?

Yes

Are sufficient details of the methods provided to allow replication by others?

Yes

Are the datasets clearly presented in a useable and accessible format?

Yes

Reviewer Expertise:

Pelvic floor disorders

I confirm that I have read this submission and believe that I have an appropriate level of expertise to confirm that it is of an acceptable scientific standard.

# Reviewer report: Reviewer response for version 2

**DOI:** 10.21956/hrbopenres.15102.r36483

**Published:** 2023-10-13

**Authors:** Sara Cichowski

**Affiliations:** 1 Oregon Health & Sciences, Portland, Oregon, USA

This is a prospective study to develop a patient-friendly website for women with pelvic organ prolapse.

 Background: no concerns

 Aim: No concerns

 Study Design:

 My primary concern is the anticipated recruitment (n=10) for the patient focus group. POP is experienced and impacts life in unique ways. Additionally the information a young, premenopausal woman needs/wants may vary from an elderly, post-menopausal woman. Likewise there may be differing cultural, contextual beliefs that require specific educational needs. I think the anticipated sample size is well below what will be needed to make this website applicable to a generalizable population. Even in qualitative approaches there can be power estimations for thematic saturation. Do the researchers have a group size estimate for thematic saturation? Would the authors considers creating a table of intentional sampling? Usually for qualitative research it's not a random pick but an intentional selection so that a broad, diverse group is selected ideally representing many viewpoints/needs/perspectives.  I would consider intentionally sampling based on age, desire for future childbearing, menopausal status, overall health status, prior experience versus no prior experience with POP, etc. This intentional sample should be described, well thought out and based on current evidence about varying populations with POP.

Is the study design appropriate for the research question?

Yes

Is the rationale for, and objectives of, the study clearly described?

Yes

Are sufficient details of the methods provided to allow replication by others?

Yes

Are the datasets clearly presented in a useable and accessible format?

Yes

Reviewer Expertise:

Pelvic floor disorders

I confirm that I have read this submission and believe that I have an appropriate level of expertise to confirm that it is of an acceptable scientific standard, however I have significant reservations, as outlined above.

# Response

**Published:** 2024-01-03

**Authors:** Louise Carroll

**Affiliations:** Physiotherapy, University College Dublin, Dublin, Leinster, Ireland

**My primary concern is the anticipated recruitment (n=10) for the patient focus group. POP is experienced and impacts life in unique ways. Additionally the information a young, premenopausal woman needs/wants may vary from an elderly, post-menopausal woman. Likewise there may be differing cultural, contextual beliefs that require specific educational needs. I think the anticipated sample size is well below what will be needed to make this website applicable to a generalizable population.**

 The authors’ previous work has been specifically focused on younger premenopausal women with POP. This research aims to build on these findings, in particular the recommendations of this cohort with respect to information needed. The authors agree that the information needs of a younger premenopausal woman are likely to differ from those of older post-menopausal women. Therefore the authors intend to focus on information provision primarily for this younger premenopausal cohort. We envisage carrying out future research with the aim of further developing the website to meet the requirements of a broader population of women e.g. older women, women from specific minority populations.

**Even in qualitative approaches there can be power estimations for thematic saturation. Do the researchers have a group size estimate for thematic saturation? Would the authors considers creating a table of intentional sampling? Usually for qualitative research it's not a random pick but an intentional selection so that a broad, diverse group is selected ideally representing many viewpoints/needs/perspectives.  I would consider intentionally sampling based on age, desire for future childbearing, menopausal status, overall health status, prior experience versus no prior experience with POP, etc. This intentional sample should be described, well thought out and based on current evidence about varying populations with POP.**

 Thank you for this valuable suggestion. As suggested, the authors have created a sampling table to ensure women with a range of prolapse stages, ages, parity and desire for no or more pregnancies are represented in the sample. Further recruitment is ongoing to ensure saturation is reached across all categories.

# Reviewer report: Reviewer response for version 2

**DOI:** 10.21956/hrbopenres.15102.r36210

**Published:** 2023-10-13

**Authors:** Rohna Kearney, Rebecca Helliwell

**Affiliations:** 1 Manchester University NHS Foundation Trust, Manchester, UK; 2 Gynaecology, Manchester University NHS Trust, Manchester, Lancashire, UK

This is a well written study protocol to co-design a website with patients and healthcare providers to provide information on pelvic organ prolapse for women. The case for  website creation is well made.

 Women will be recruited from an existing online forum. Previous work published from this online group, referenced in this protocol, reported an age range of 32-41 years. All women included in that study (reference 6) were reported of fair to good health. How will the researchers ensure the study produces an online resource that also meets the needs of older women, those less engaged with internet and online media and those with medical co-morbidities? If the focus is on information provision for premenopausal women, this should be explicitly stated.

 As 10 women will be recruited, how will the authors ensure appropriate representation of the population of women with prolapse. Will the website be accessible to women who do not speak English? Is 10 women sufficient for theme saturation?

 I suggest discussing the strengths and limitations of the approach proposed.

 It would be helpful to reference the HSE/NALA guidelines discussed for readers not familiar with these.

Is the study design appropriate for the research question?

Partly

Is the rationale for, and objectives of, the study clearly described?

Yes

Are sufficient details of the methods provided to allow replication by others?

Yes

Are the datasets clearly presented in a useable and accessible format?

Not applicable

Reviewer Expertise:

Urogynaecology, pelvic floor, self-management, pelvic floor imaging, obstetric childbirth injury

We confirm that we have read this submission and believe that we have an appropriate level of expertise to confirm that it is of an acceptable scientific standard, however we have significant reservations, as outlined above.

# Response

**Published:** 2024-01-03

**Authors:** Louise Carroll

**Affiliations:** Physiotherapy, University College Dublin, Dublin, Leinster, Ireland

**How will the researchers ensure the study produces an online resource that also meets the needs of older women, those less engaged with internet and online media and those with medical co-morbidities? If the focus is on information provision for premenopausal women, this should be explicitly stated.**

 The authors’ previous work has been specifically focused on younger premenopausal women with pelvic organ prolapse (POP). This research aims to build on these findings, in particular the recommendations of this cohort with respect to information needed.

 The literature to date shows that POP symptom bother is not always explained by level of anatomic prolapse. In our previous work (Carroll et al 2023) and in other research, it has been suggested that the aetiology of POP in younger women differs from that in older women, in whom POP symptoms tend to more closely reflect anatomic POP (Alcalay 2015, Rinne 1999).

 This younger cohort tend to report more symptom bother which in many cases levels off around menopause (Miedel et al 2008). Central sensitization and/or myofascial pain have been proposed as a possible explanation for this, with Vij et al finding a 30% rate of central sensitization in a sample of women with POP from a general GOPD (Vij et al 2019, Dixon et al 2019, Adams et al 2014).

 The prevalence of POP in premenopausal women in Ireland and elsewhere is uncertain; however Durnea et al reported a 90% incidence of anatomic POP in primparous women 1 year after childbirth. 20% of these women were symptomatic.

 Research to date has found that neuroscience education, cognition-targeted exercise therapy, sleep management, stress management, and/or dietary intervention to be important facets for the treatment of central sensitization (Nijs et al 2019). If central sensitization is an important factor, it would be interesting to see how a biopsychosocial approach to POP including the information proposed through this intervention would affect experience of this cohort of women with POP.

 Therefore the authors intent is to focus on information provision for premenopausal women primarily. This has been clarified in the article.

_Carroll L, Sullivan CO, Doody C, Perrotta C, Fullen BM. Pelvic organ prolapse: Women’s experiences of Accessing Care & Recommendations for improvement. BMC Women's Health. 2023 Dec;23(1):1-3._

_Alcalay M, Stav K, Eisenberg VH. Family history associated with pelvic organ prolapse in young women. International urogynecology journal. 2015 Dec;26:1773-6._

_Rinne KM, Kirkinen PP. What predisposes young women to genital prolapse?. European Journal of Obstetrics & Gynecology and Reproductive Biology. 1999 May 1;84(1):23-5._

_Miedel A, Tegerstedt G, Mörlin B, Hammarström M. A 5-year prospective follow-up study of vaginal surgery for pelvic organ prolapse. International Urogynecology Journal. 2008 Dec;19:1593-601._

_Vij M, Davies A, Dua A, Freeman R. The proportion of women with central sensitivity syndrome in gynecology outpatient clinics (GOPDs). International Urogynecology Journal. 2019 Mar 4;30:483-8._

_Dixon AM, Fitzgerald CM, Brincat C. Severity and bother of prolapse symptoms in women with pelvic floor myofascial pain. International Urogynecology Journal. 2019 Nov;30:1829-34._

_Adams K, Osmundsen B, Gregory WT. Does fibromyalgia influence symptom bother from pelvic organ prolapse?. International urogynecology journal. 2014 May;25:677-82._

_Durnea CM, Khashan AS, Kenny LC, Durnea UA, Smyth MM, O’Reilly BA. Prevalence, etiology and risk factors of pelvic organ prolapse in premenopausal primiparous women. International urogynecology journal. 2014 Nov;25:1463-70._

_Nijs J, Leysen L, Vanlauwe J, Logghe T, Ickmans K, Polli A, Malfliet A, Coppieters I, Huysmans E. Treatment of central sensitization in patients with chronic pain: time for change?. Expert Opinion on Pharmacotherapy. 2019 Nov 2;20(16):1961-70._

**As 10 women will be recruited, how will the authors ensure appropriate representation of the population of women with prolapse?**

 The authors acknowledge that representation of the population of all women with POP will not be fully addressed as focus of the authors research is on the impact of POP on premenopausal women. The authors will use a sampling grid to ensure recruitment of pre-menopausal women with stages I-IV prolapse, of varying parity and those who do and do not desire more children.  To increase impact and applicability, we would envisage after the website is complete that more research will be carried out to further develop the website with the requirements of a broader population of women e.g. older women, women from specific minority populations including a broader range of recruitment strategies taken into account.

**Will the website be accessible to women who do not speak English?**

 The website will be developed in English but will be enabled to allow auto-translation to multiple languages.

**Is 10 women sufficient for theme saturation?**

 The authors have created a sampling table to ensure women with a range of prolapse stages, ages, parity and desire for no or more pregnancies are represented in the sample. Further recruitment is ongoing to ensure saturation is reached across all categories.

**It would be helpful to reference the HSE/NALA guidelines discussed for readers not familiar with these.**

 The authors have added this information to the reference section.

# Reviewer report: Reviewer response for version 1

**DOI:** 10.21956/hrbopenres.15032.r35508

**Published:** 2023-08-18

**Authors:** Louise Brennan

**Affiliations:** 1 Discipline of Physiotherapy, School of Medicine, Trinity College Dublin, Dublin, Ireland

A well-written, detailed protocol for the co-design of a website for women with pelvic organ prolapse. The introduction clearly describes the problem the website aims to solve and this is important work which will support an under-served population. The methodology is guided by a very appropriate framework (WDMHC) which will enable comprehensive input from stakeholders and should result in a highly user-friendly website.

 Is the context for this website local, national or international? Providing some context for this will help provide background to the study for the reader, will guide website design and will help identify if participant representatives in focus groups appropriately represent target population. If the website is for a national / local audience, consider including appropriate references re health literacy (e.g. HSE/NALA).

 Focus groups: Can you report why participants with advanced POP are not included in the focus groups? Family members and spouses will provide insightful input to this project - could they be included at some stage? Will the website developer attend any part of these sessions?

 Heuristics will ideally be assessed by someone external to the development team - could a patient rep and HCP complete this assessment also? Will you make further adjustments to the website based on the feedback from content-based testing?

Is the study design appropriate for the research question?

Yes

Is the rationale for, and objectives of, the study clearly described?

Yes

Are sufficient details of the methods provided to allow replication by others?

Yes

Are the datasets clearly presented in a useable and accessible format?

Not applicable

Reviewer Expertise:

Digital health, website co-design, physical rehabilitation

I confirm that I have read this submission and believe that I have an appropriate level of expertise to confirm that it is of an acceptable scientific standard.

# Response

**Published:** 2023-08-30

**Authors:** Louise Carroll

**Affiliations:** Physiotherapy, University College Dublin, Dublin, Leinster, Ireland

Dear Dr. Brennan,

 Thank you very much for your time and expertise in reviewing our study protocol. We have addressed your feedback below and also in the updated version of the study article.

 Kind regards,

 Louise Carroll, Brona Fullen, Catherine Doody, Cliona O’ Sullivan & Carla Perrotta

**Is the context for this website local, national or international? Providing some context for this will help provide background to the study for the reader, will guide website design and will help identify if participant representatives in focus groups appropriately represent target population. If the website is for a national / local audience, consider including appropriate references re health literacy (e.g. HSE/NALA).**

 The context for this website is national. As such, we will consult the NALA ‘Literacy audit for healthcare settings’ and HSE ‘Guidelines for Communicating Clearly using Plain English with our Patients and Service Users’ to inform the design.

**Focus groups: Can you report why participants with advanced POP are not included in the focus groups?**

 We have amended the study inclusion/exclusion criteria to include women with Stage IV POP. As many women have corrective surgery as a matter of urgency with this level of advanced POP and as it is relatively rare, recruitment of women with Stage IV POP would be challenging; to date we do not have anyone with Stage IV POP enrolled in the study.

**Family members and spouses will provide insightful input to this project - could they be included at some stage?**

 For the current project we have no plan to include family members and spouses; however we may explore this perspective in future work.

**Will the website developer attend any part of these sessions?**

 Yes, the developer will attend both the patient and HCP workshops.

**Heuristics will ideally be assessed by someone external to the development team - could a patient rep and HCP complete this assessment also?**

 We have amended the study methodology to reflect this recommendation.

**Will you make further adjustments to the website based on the feedback from content-based testing?**

 Yes the website will be adjusted based to content-based testing feedback.
